# Building Large-Scale Quantitative Imaging Databases with Multi-Scale Deep Reinforcement Learning: Initial Experience with Whole-Body Organ Volumetric Analyses

**DOI:** 10.1007/s10278-020-00398-y

**Published:** 2021-01-19

**Authors:** David J. Winkel, Hanns-Christian Breit, Thomas J. Weikert, Bram Stieltjes

**Affiliations:** Department of Radiology, University Hospital of Basel, Basel, Basel-Stadt Switzerland

**Keywords:** Tomography, X-ray computed, Organ size, Workflow, Database, Deep learning

## Abstract

To explore the feasibility of a fully automated workflow for whole-body volumetric analyses based on deep reinforcement learning (DRL) and to investigate the influence of contrast-phase (CP) and slice thickness (ST) on the calculated organ volume. This retrospective study included 431 multiphasic CT datasets—including three CP and two ST reconstructions for abdominal organs—totaling 10,508 organ volumes (10,344 abdominal organ volumes: liver, spleen, and kidneys, 164 lung volumes). Whole-body organ volumes were determined using multi-scale DRL for 3D anatomical landmark detection and 3D organ segmentation. Total processing time for all volumes and mean calculation time per case were recorded. Repeated measures analyses of variance (ANOVA) were conducted to test for robustness considering CP and ST. The algorithm calculated organ volumes for the liver, spleen, and right and left kidney (mean volumes in milliliter (interquartile range), portal venous CP, 5 mm ST: 1868.6 (1426.9, 2157.8), 350.19 (45.46, 395.26), 186.30 (147.05, 214.99) and 181.91 (143.22, 210.35), respectively), and for the right and left lung (2363.1 (1746.3, 2851.3) and 1950.9 (1335.2, 2414.2)). We found no statistically significant effects of the variable contrast phase or the variable slice thickness on the organ volumes. Mean computational time per case was 10 seconds. The evaluated approach, using state-of-the art DRL, enables a fast processing of substantial amounts irrespective of CP and ST, allowing building up organ-specific volumetric databases. The thus derived volumes may serve as reference for quantitative imaging follow-up.

## Introduction

Accurate whole-body organ volumetric analyses could have a substantial impact on clinical practice. Areas of application include, but are not limited to, imaging of patients with chronic hepatitis [1], nonalcoholic fatty liver disease [2], acute liver failure [3], change in kidney volume after kidney transplant [4], assessing splenomegaly [5] , or assessing lung volumes after reduction for emphysema [6] . Another important application is the assistance in surgical planning, e.g., preoperative analysis of liver volumes before transplantation [7] . Organ volumes, however, vary with age, sex, weight, and height. Therefore, reference datasets of normal organ volumes for patients with different physical conditions based on sufficiently large cohort of normal patients are needed. The creation of such organ-specific databases for whole-body organs is a currently an unsolved task; one of the main reasons is that manual organ segmentation is a time-consuming task that is difficult to incorporate in high-throughput clinical routine [8]. Furthermore, it is prone to a relevant amount of inter- and intra-reader variability [9].

A potential solution is the use of artificial intelligence, more precisely deep reinforcement learning (DRL) using 3D landmark detection [10]. With this technology, whole body organ volumetric analyses can be derived in a short amount of time. Furthermore, applying this to liver volumetric analyses has recently been shown to yield an excellent agreement with human readers [9].

We hypothesize that the approach is capable of building databases that can assist reporting. Given the availability of enough input data, the workflow would easily enable to create norm-based values and based on that, automated, disease-related outlier detection would be possible. In order to illustrate the suggested workflow, we performed an analysis of 431 multiphasic computer tomography (CT) datasets, including 10,508 organ volumes using DRL.

## Materials and Methods

The local ethics committee (Northwest and Central Switzerland; EKNZ 2019-00634) approved the study.

### Case Selection

Using an in-house-developed Radiology Information System/Picture Archiving and Communication System (RIS/PACS) search engine, we identified all multiphasic abdominal CTs in an adult population performed with the clinical suspicion for an intestinal bleeding in a time range from 11/2012 to 01/2019. We selected this specific cohort as the underlying CT protocol at our institution remained unchanged during this time period and included a multiphasic scan protocol with the following items: a non-contrast (nc), arterial (art), and portalvenous (pv) contrast phase (CP) with axial reconstruction in both 1.5-mm and 5-mm slice thickness (ST), totaling six series per patient. Using this protocol allowed us to investigate the influence of CP and ST on the outputted organ volume.

The initial search resulted in 759 CT scans. During the download process, the data was completely anonymized. Patients were only included in the evaluation if nc, art, and pv phases with both 1.5-mm and 5-mm slice thickness reconstructions (for the abdomen) and—if available—pv series covering the lung in 5-mm slice thickness were available, and the parenchyma of all organs of interest was covered on all series. After manual identification, 438 patients fulfilled all criteria. In 165 of those 431 cases, the thorax was included in pv phase only, as thoracic imaging was only performed in the presence of a potential, concomitant supradiaphragmal pathology.

### Multiphasic CT Examination

The selected abdominal examinations were performed on three different CT scanners: Somatom Definition FLASH (Siemens Healthineers, Erlangen, Germany), Somatom Definition AS + (Siemens Healthineers, Erlangen, Germany), and Somatom Sensation (Siemens Healthineers, Erlangen, Germany) with 2 × 128, 128, and 16 slices, respectively. Iterative reconstruction kernels—I30 and I70—were applied. All patients received a weight-adapted (1–1.5 ml/kg body weight) iodine-based contrast agent injection (Iopromide, generic name: Ultravist 370, Bayer HealthCare Pharmaceuticals, Berlin, Germany).

### Automated Volumetric Analyses

**Fig. 1:**
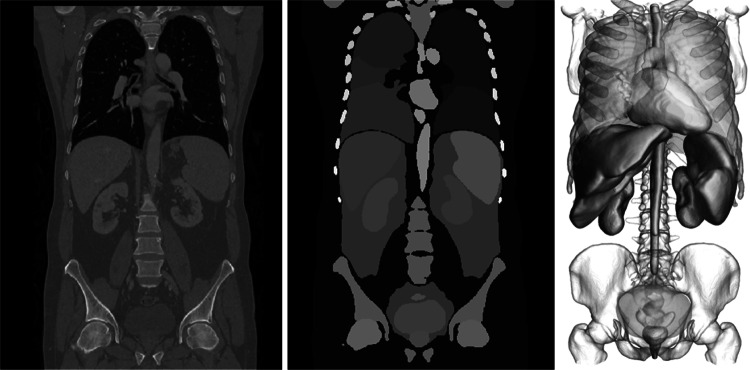
Visualization of the deep reinforcement learning workflow with the CT image data—here in portal venous phase and 5-mm slice thickness—on the left side. The image is then processed by the DRL algorithm, involving the volumetric parsing of the organs of interest. This subsumes the localization of key anatomical landmark points that isolate each organ within the image, followed by the surface segmentation (middle image). In a next step, all masks from all organs can be used to provide three dimensional models of the organs or to calculate organ volumes in milliliter

The abdominal volumetric analyses were calculated with a prototype, non-commercially available software (NeuronX, Siemens Healthineers, Erlangen, Germany). The first step involves the volumetric parsing of the organs of interest. This subsumes the localization of key anatomical landmark points that isolate each organ within the image, followed by the surface segmentation (see Fig. 1).

The multi-scale deep reinforcement learning framework [11] is used for the automatic detection of anatomical landmarks. Within this framework, the localization of an arbitrary anatomical landmark is formulated as a navigation problem for an artificial neural agent within the scale-space (i.e., the discrete multi-scale representation) of the image. Using state-of-the-art convolutional neural network architectures and elements of reinforcement learning [12], one can learn an effective strategy that drives the navigation of the agent from any arbitrary position in the image to the position of a landmark of interest. In practice, the navigation process across the image scale-space is very efficient, enabling a real-time detection of the organs of interest on the high-resolution, volumetric images. To ensure the robustness of the navigation, a robust statistical shape model is estimated and used to constrain the navigation of individual agents such that the distribution of the detected points is consistent with the prior knowledge of the distribution of anatomical structures in the human body [13].

Based on the extracted landmarks, the local image region around each organ was isolated/cropped and used to drive the surface segmentation. Whole-body organ segmentations were performed using a deep image-to-image neural network with adversarial training. The approach comprises training a neural network with an encoder-decoder architecture with multilevel feature concatenation to generate segmentations that are spatially accurate and difficult to distinguish from manual segmentations [14] . Based on the extracted segmentation for each organ of interest in a volumetric mask representation format, organ volumes (in milliliter) were automatically calculated and the computational time per case was recorded. The algorithm was trained for whole-body organ volumetry on an independent sample of approximately 5000 whole-body CT datasets. The analyses have been performed on a commercially available laptop with an Intel ® Core™ CPU i7-8850H at 2.60 GHz with integrated Intel ® UHD graphics.

### Manual Contour Segmentation for Outlier Validation

In order to validate the segmentation results beyond the result of previous studies, we analyzed outliers from the automatically calculated organ volumes. Here, a radiology resident (PGY-4, D.J.W.) performed a manual contour segmentation with shape interpolation [8] using a commercially available software solution (syngo.via VB30A, MM Reading Workflow, Siemens Healthineers, Erlangen, Germany) on a single outlier case for all organs by random selection of a case in the lowest and highest 10% of the respective organ volume. In a next step, we used the manually derived organ volume for a head-to-head comparison with the automatically derived organ volumes from the software solution in terms of absolute and relative value comparison.

### Statistical Testing

Variations of volumes between the multiphasic series of the abdominal organs were assed using a repeated measure analysis of variance (ANOVA) with the different contrast-phases as the between-group factor and the slice thickness reconstruction as the within-subject factor. We especially controlled for between-participant variation over all our within-subjects variables.

All statistical calculations and graphical analyses were performed using R (R Version 3.6.0, R Core Team (2019). R: A language and environment for statistical computing. R Foundation for Statistical Computing, Vienna, Austria. URL, https://www.R-project.org/). *p* < 0.05 were considered statistically significant. To adjust for multiple testing and to control the type I error in our study, we performed a Bonferroni correction of the significance level of the individual test with the following formula: $p* = \frac{\alpha }{\eta }$, where *α* is the critical *p* value, and *η* is the number of comparisons.

## Results

**Fig. 2:**
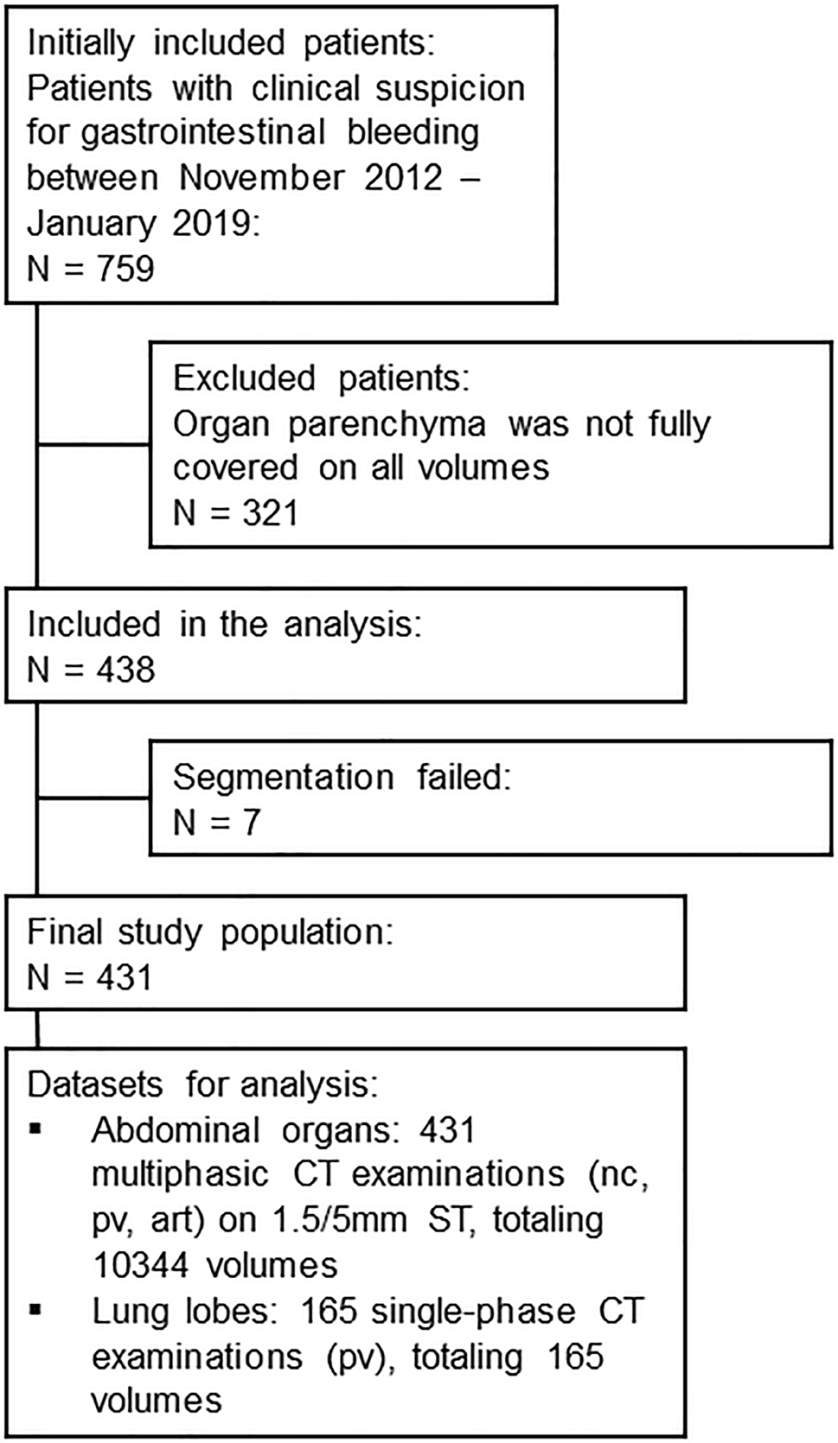
Flowchart outlines the selection of the final study population with inclusion and exclusion criteria within the defined observation period. nc non-contrast, pv portal venous, art arterial

**Fig. 3:**
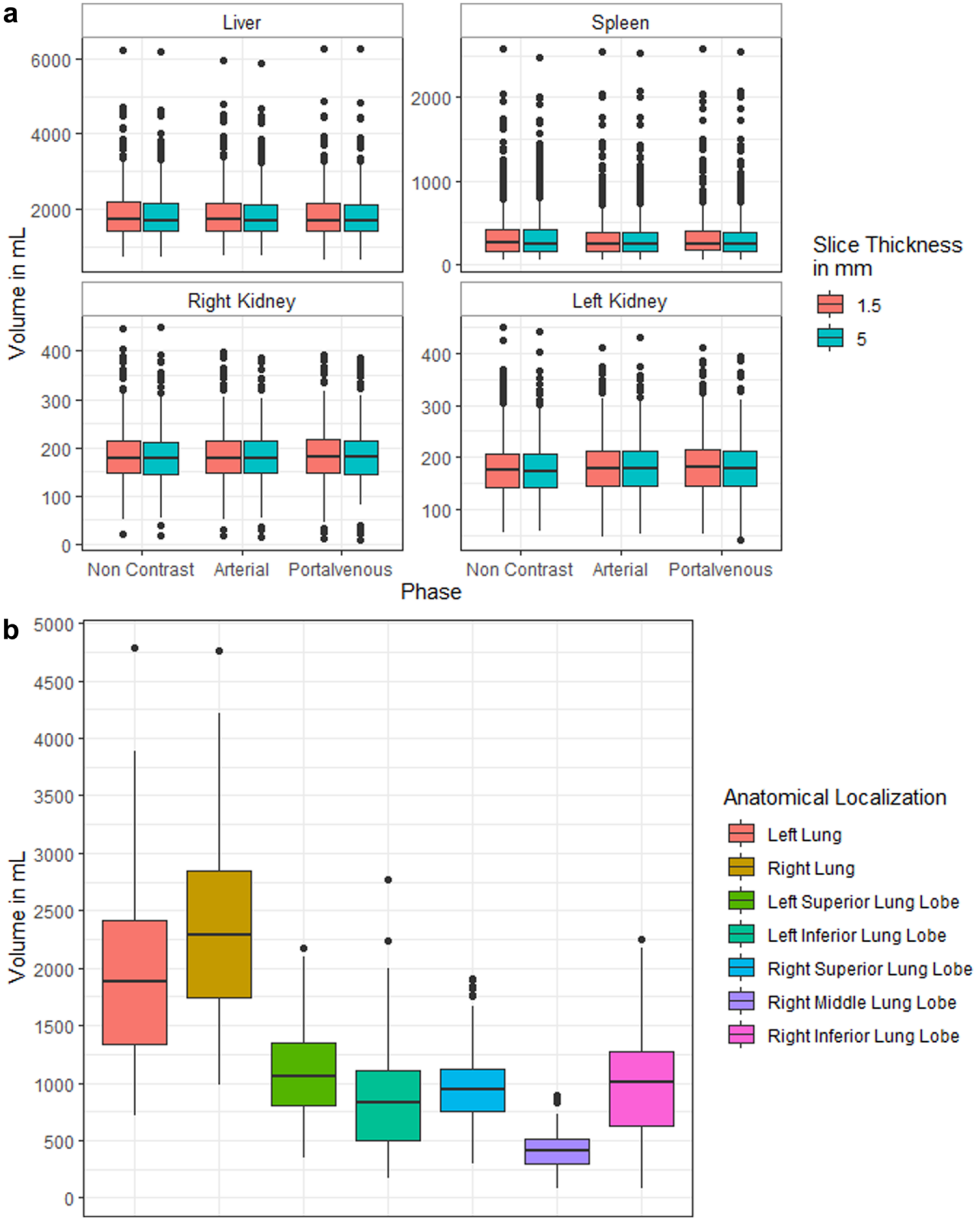
Quantitative evaluation of the abdominal organs (**a**) including the liver, spleen and both kidneys and the lung lobes and their respective sub lobes (**b**). For the abdominal organs, the *x*-axis displays the different contrast phases, and the *y*-axis represents the organ volume in milliliter. Different slice thickness measurements are color encoded. For the thoracis lung lobes, the measurements displayed were acquired using portal venous images only. The anatomical reference of the lobes and sub lobes in color encoded

After manual identification, 438 patients fulfilled the inclusion criteria. The algorithm failed to process the data in seven cases. Therefore, we included the results of 431 patients. This led to an inclusion of 10,508 organ volumes in total for the analysis with (see Fig. 2). A detailed summary of the automatically computed whole-body organ volumes can be found in Table 1 and Table 2—including detailed information on CP and ST—and is illustrated in Fig. 3. A detailed summary of the repeated ANOVA statistics can be found in Table 3.

**Table 1:** Abdominal organ volumes. Measurements of organ volumes for abdominal sub-categories depending on contrast phase and slice thickness. Values given represent volumes in milliliter. SD = standard deviation

	Contrast phase	Slice thickness	Mean ± SD	Interquartile range (1^st^ Qu, 3^rd^ Qu)	Range (min–max)
Abdomen (*n* = 431 for each sub-category)					
Liver	Non contrast	1.5	1893.5 ± 684	1437.7, 2185.5	753.2, 6203.2
	Non contrast	5	1864.0 ± 680	1411.6, 2154.6	733.5, 6200.0
	Arterial	1.5	1881.4 ± 661	1443.2, 2182.8	786.2, 5944.6
	Arterial	5	1851.3 ± 656	1414.2, 2144.5	778.9, 5881.4
	Portal venous	1.5	1875.8 ± 660	1449, 2162	641.6, 6278
	Portal venous	5	1868.6 ± 667	1426.9, 2157.8	640.4, 6278.4
Spleen	Non contrast	1.5	363.16 ± 363	166.01, 412.90	45.46, 2593.04
	Non contrast	5	361.38 ± 361	162.44, 418.48	57.48, 2481.20
	Arterial	1.5	343.92 ± 343	160.90, 380.48	54.73, 2557.53
	Arterial	5	339.97 ± 339	157.16, 385.34	50.31, 2528.89
	Portal venous	1.5	348.1 ± 348	168.8, 397.7	50.5, 2577.4
	Portal venous	5	350.19 ± 318	45.46, 395.26	45.46, 2593.04
Right kidney	Non contrast	1.5	187.33 ± 59	147.04, 215.65	21.17, 445.51
	Non contrast	5	184.94 ± 58	145.25, 212.02	19.27, 450.20
	Arterial	1.5	186.97 ± 59	148.88, 213.64	17.07, 397.83
	Arterial	5	185.19 ± 58	147.41, 213.30	15.14, 387.60
	Portal venous	1.5	187.83 ± 57	147.59, 218.65	13.03, 391.29
	Portal venous	5	186.30 ± 58	147.05, 214.99	10.05, 214.99
Left kidney	Non contrast	1.5	181.27 ± 56	142.51, 206.81	55.73, 450.78
	Non contrast	5	179.32 ± 55	141.52, 205.46	56.93, 442.05
	Arterial	1.5	182.78 ± 55	143.31, 212.15	45.43, 412.13
	Arterial	5	181.66 ± 54	143.32, 211.09	51.31, 431.29
	Portal venous	1.5	184.21 ± 56	145.83, 213.92	50.54, 411.77
	Portal venous	5	181.91 ± 55	143.22, 210.35	41.58, 450.78

**Table 2:** Lung volumes. Measurements of organ volumes for thoracic sub-categories in portal venous phase only. Values given represent volumes in milliliter. SD = standard deviation

	Mean ± SD	Interquartile range (1^st^ Qu, 3^rd^ Qu)	Range (min–max)
Thorax (*n* = 164 for each sub-category)			
Left lung	1950.9 ± 763	1335.2, 2414.2	711.3, 4787.3
Right lung	2363.1 ± 757	1746.3, 2851.3	982.3, 4760.2
Left superior lung lobe	1089.0 ± 395	799.9, 1346.5	344.7, 2168.0
Left inferior lung lobe	861.9 ± 444	502.1, 1108.1	172.6, 2772.4
Right superior lung lobe	962.9 ± 327	754.3, 1122.4	291.8, 1908.1
Right middle lung lobe	414.27 ± 152	294.95, 507.54	82.52, 896.16
Right inferior lung lobe	985.87 ± 414	631.46, 1271.94	87.42, 2249.81

**Table 3:** Results of repeated measures ANOVA for abdominal organs. Values representing *p* values

Organ	Contrast phase*	Slice thickness	Combination
Liver	0.893	0.595	0.999
Spleen	0.775	0.951	1.000
Right kidney	0.884	0.621	0.984
Left kidney	0.978	0.731	0.987

^*^Bonferroni adjusted

### Organ Volumes on Non-Contrast Phase Series

The volumes computed by the algorithm for the abdominal organs were (mean ± SD, in milliliter, 5 mm ST): 1864.0 ± 680 for the liver, 361.38 ± 361 for the spleen, 184.94 ± 58 for the right kidney, and 179.32 ± 55 for the left kidney.

### Organ Volumes on Arterial Phase Series

The volumes computed by the algorithm for the abdominal organs were (mean ± SD, in milliliter, 5 mm ST): 1851.3 ± 656 for the liver, 339.97 ± 339 for the spleen, 185.19 ± 58 for the right kidney, and 181.66 ± 54 for the left kidney.

### Organ Volumes on Portal-Venous Phase Series

The volumes computed by the algorithm for the abdominal organs were (mean ± SD, in milliliter, 5 mm ST): 1868.6 ± 667 for the liver, 350.19 ± 318 for the spleen, 186.30 ± 58 for the right kidney, and 181.91 ± 55 for the left kidney. The volumes computed by the algorithm for the lung lobes and sub lobes were (mean ± SD, in milliliter): 1950.9 ± 763 for the left lung lobe, 2363.1 ± 757 for the right lung lobe, 1089.0 ± 395 for the left superior lung lobe, 861.9 ± 444 for the left inferior lung lobe, 962.9 ± 327 for the right superior lung lobe, 414.27 ± 152 for the right middle lung lobe, and 985.87 ± 414 for the right inferior lung lobe.

### Results from Repeated Measure ANOVA

We found no significant effects of the between-group variable contrast phase neither of the within-subject variable slice thickness nor of the combination of those factors on the automatically computed abdominal organ volumes.

### Quantitative Outlier Validation

**Fig. 4:**
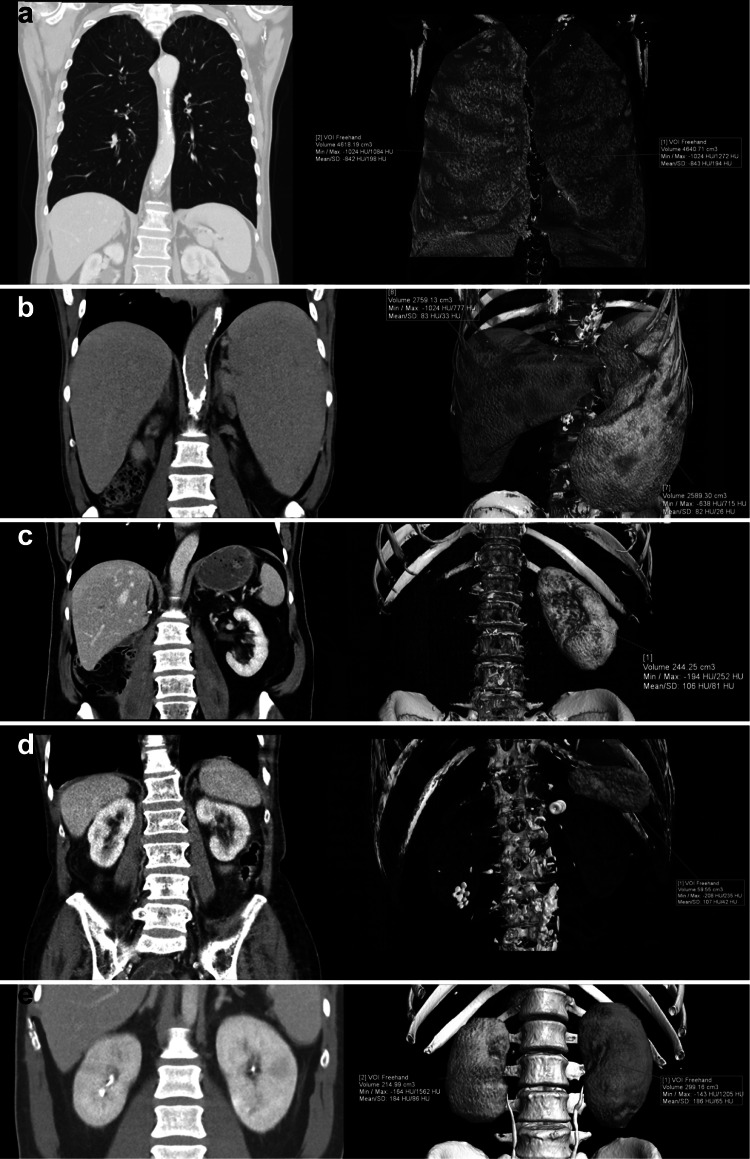
(**a**) Exemplary case of a 48-year-old patient with known obstructive lung disease and emphysema. The CT examination was scheduled with the suspicion of a rectal bleeding. The manual contour segmentation with shape interpolation yielded a left lung volume of 4640 ml and a right lung volume of 4618 ml. NeuronX calculated a left lung volume of 4787 ml (+ 147 ml) and a right lung volume of 4760 ml (+ 142 ml). (**b**) Exemplary case of a 62-year-old patient with a myeloproliferative disease and a known hepato- and splenomegaly. The CT examination was scheduled due to a suspected spleen rupture. The manual contour segmentation with shape interpolation yielded a liver volume of 2759 ml and a spleen volume of 2589 ml. NeuronX calculated a liver volume of 2812 ml (+ 53 ml) and a spleen volume of 2608 (+ 19 ml). (**c**) Exemplary case of a 67-year-old patient with a right kidney resection due to a sarcoma. The CT examination was scheduled due to a suspected bleeding in the postoperative bed. The manual contour segmentation with shape interpolation yielded a left kidney volume of 244 ml. NeuronX calculated a left kidney volume of 267 ml (+ 23 ml). NeuronX did not calculate any values for the missing right kidney as the 3D landmark detection algorithm did not find the object in the 3D space. The case was not included in the final analysis as it did not fulfill the inclusion criteria. (**d**) Exemplary case of a 45-year-old patient with a suspected intestinal bleeding. The manual contour segmentation with shape interpolation yielded a spleen volume of 60 ml. NeuronX calculated a spleen volume of 66 ml (+ 6 ml). **e** Exemplary case of a 32-year-old patient with a suspected intestinal bleeding. The examination revealed a pyelonephritis affecting both kidneys. The manual contour segmentation with shape interpolation yielded a right sided kidney volume of 215 ml and a left sided kidney volume of 299 ml. NeuronX calculated a right sided kidney volume of 224 ml (+ 9 ml) and a left sided kidney volume of 315 ml (+ 16 ml)

Table 4 summarizes the outlier cases with absolute and relative differences between the automatically and manually derived volumes. Visualizations from the manual contour segmentation are shown in Fig. 4a–e.

**Table 4:** Internal outlier validation. Cases were randomly selected in the highest and lowest 10% of the respective organ volumes. For paired organs (lungs, kidneys), the mean values for both organs are shown

Organ	Underlying pathology	Volume spectrum	Automatically derived results (in milliliter)	Manually derived results (in milliliter)	Absolute difference (in milliliter)	Relative difference (in percent)
Lung	Obstructive lung disease	High	4773	4629	+ 144	3
	Volume reduction	Low	930	892	+ 38	4
Liver	Acute viral hepatitis	High	4562	4392	+ 170	4
	Liver resection	Low	791	743	+ 48	6
Spleen	Myeloproliferative disease	High	2608	2589	+ 19	1
	None	Low	66	60	+ 6	10
Kidney	Pyelonephritis	High	269	256	+ 13	5
	Nephrectomy	Low	0	0	0	0

### Processing Time

The total processing time for all 10,508 volumes was 1 h, 11 min, and 40 s representing a computational time per case of 9.94 s and a computational time per volume of 0.9 s.

## Discussion

Organ volumetric analyses have the capability to provide meaningful information for the referring physician. Areas of application range from the assessment of absolute organ volumes [5, 15] to treatment monitoring [4, 16]. Furthermore, a norm collective considering basic patient characteristics (e.g., sex, age) allows a differentiation of normal from pathologic organ volumes. As an example, Kawel-Böhm et al. investigated reference values for morphologic and function cardiac MRI parameters adjusted for sex and age [17]. To date, no such analyses exist for other organs.

One reason for the lack of such studies is certainly that organ volumetric analyses have been performed in the past using manual contour segmentation, partially employing techniques to speed up the process, such as semi-automated contour interpolation [8, 9]. Nevertheless, in order to create a meaningful reference database for organ volumes, the number of cases that needs to be processed would exceed manual segmentation capabilities. Here, AI systems are potentially useful to extract information from large-scale populations with the goal of building databases for that in turn can be used in order to assist reporting with real-time inference [18]. In order to ultimately reach this goal, fully automated quantification pipelines are required to collect patient measurements across a large-scale population. In particular, the combination of convolutional neural networks (CNN) and reinforcement learning (RL), known under the term deep reinforcement learning (DRL) [19], has proven to be suitable to autonomously support clinical decision making.

Using DRL to fill this gap in clinical practice has one prerequisite, namely, that the outputted values are correct and that outliers on both sides—including missing organ—are accurately captured and make sense. As we did not perform a dedicated comparison between the automatically and manually derived organ volumes, serving as a reference standard, we developed two strategies in order to review the usefulness and correctness of our values: (i) referencing our organ volumes to values published in the literature, except for liver volumes, since previous work [9], using the same framework, showed an excellent agreement between the averaged liver volumes of three human readers and the AI approach and (ii) internally validating outlier cases from the AI solution by comparison with manual contour segmentation.

Concerning splenic organ volumes, normal CT values in the literature range from mean volumes of 214.6 cm^3^ (range 107.2 to 314.5 cm^3^) [20] to 127.4 ± 62.9 cm^3^ (range: from 22 to 417 cm^3^) [21]. We computed a mean value of 350.19 ± 318 cm^3^ (range 45.46 to 2593.04 cm^3^). However, some of the patients included in our study had underlying diseases, which caused a splenomegaly, explaining the wide range in our study. The algorithm, nevertheless, was able to capture these “real” high outliers (see Fig. 4b) or—alternatively—very low spleen volumes (see Fig. 4d). Concerning kidney volumes, normal values in the literature, for example evaluated with magnetic resonance imaging (MRI) have been reported to range from 202 ± 36 cm^3^ for men to 154 ± 33 cm^3^ for women [22]. Our values, however evaluated with CT, were 186.30 ± 58 cm^3^ for the right kidney and 181.91 ± 55 cm^3^ for the left kidney and are in concordance with reported values. In one case, which was not included in the final analysis, one kidney was missing due to a nephrectomy. The algorithm was able to capture this anatomical anomaly and did not compute any values (see Fig. 4c). Concerning normal lung volumes, reported values, evaluated with CT, were 2414 ± 480 cm^3^ for the left lung volume and 2869 ± 506 cm^3^ for the right lung volume [23]. The values in our cohort were 1950.9 ± 763 cm^3^ for the left lung and 2363.1 ± 757 cm^3^ for the right lung and apparently lie within the reported range. However, some values in our lung volume analysis were unexpectedly high and low. These values turned out to be due to underlying pathologies and therefore “true” outliers, as shown in and example case (Fig. 4a).

The deep reinforcement learning framework used in this study has been developed in order to enable a robust and fast detection of anatomical structures, which are a prerequisite for creating such databases in a short amount of time with excellent agreement between human readers and the algorithm. Furthermore, we demonstrated that all tested contrast phases and slice thicknesses can be used concurrently, which allows the algorithm to be used on a various set of studies. Apart from this study—to our best knowledge—no further studies have been conducted using DRL for medical image analysis for clinical purposes.

Our study has several limitations. First, we included all patients, which fulfilled the inclusion criteria, and not only healthy patients. This explains the wide range of organ volumes in our analysis. However, using the DICOM data as the visual reference, even obvious outliers in our data represented real outliers in the images in the sense of organomegaly or the opposite; this demonstrates that the algorithm is capable to process data from all patients. Second, we tested the technical feasibility of our approach without investigating if we could extract meaningful clinical data from our results. This study was supposed to build the technical groundwork for future studies on larger cohorts. Third, in seven out of 438 cases, the algorithm has not outputted values for all pairs of organs and CP and ST. In a detailed investigation, we identified a missing landmark detection for the liver parenchyma as the underlying cause. As outlined in the materials and methods section, the DRL framework presented is trained to find an object of interest using an optimal navigation path in the volumetric space. If this path is blocked due to various reasons, e.g., calcifications of the right-sided diaphragm or extensive ascites between the diaphragm and the liver, the algorithm will not detect the object and therefore not output values. Forth, the technology has not been implemented in our routine clinical workflow yet. However, we plan to implement the algorithm soon in order to build organ-specific databases that could prove useful both in the clinical routine and in research questions.

## Conclusion

In conclusion, we were able to demonstrate that the DRL framework used in this study is robust and capable to create organ-specific databases from a large population in a short amount of time. Future studies are warranted in order to apply this DRL framework on larger patient populations with dedicated statistical testing in order to evaluate the agreement between human readers and the proposed algorithm for all investigated organ. In a next step, this approach could be extended to extract organ density values, as performed in Graffy et al. [24] and automatically calculated, referenced organ volumes could enrich radiology reports.
